# Comparative Evaluation of Therapeutic Effects of Boric Acid and Triamcinolone in Nasal Polyp Culture

**DOI:** 10.1007/s12011-026-05040-6

**Published:** 2026-03-14

**Authors:** Nurullah Türe, Meliha Koldemir Gündüz, Güllü Kaymak, Ayşe Koçak Sezgin, Mehmet Varol

**Affiliations:** 1 Department of Otorhinolaryngology, Kütahya Health Sciences University, Bahçelievler Mahallesi Başyiğit Sokak Semakent Sitesi A Blok no:29/8, Kütahya, Türkiye; 2 Department of Basic Engineering Sciences, Kütahya Health Sciences University, Kütahya, Türkiye; 3 Department of Medical Services and Techniques, Kütahya Health Sciences University, Kütahya, Türkiye; 4 Department of Medical Biochemistry, Kütahya Health Sciences University, Kütahya, Türkiye; 5 Department of Otorhinolaryngology, Kütahya City Hospital, Kütahya, Türkiye

**Keywords:** Chronic rhinosinusitis, Nasal polyps, Boric acid, Triamcinolone acetonide, Oxidative stress, MMP-9, TIMP-1

## Abstract

This study aimed to elucidate the therapeutic effects of boric acid (BA) in human nasal polyp cultures, specifically investigating its ability to modulate oxidative stress, inflammation, and remodeling, and to assess its potential as an adjuvant to triamcinolone acetonide (TA). Nasal polyp tissues from 30 patients with CRSwNP, obtained prior to functional endoscopic sinus surgery, were cultured ex vivo for 72 h. Within-patient randomized groups were: control, BA 2.5 mM, TA 10 µM, BA 2.5 mM + TA 10 µM, and BA 2.5 mM + TA 5 µM. At the endpoint, cytokines (IL-2, IL-4, IL-5, IL-13, TNF-α) and MMP-9/TIMP-1 (ELISA/RT-qPCR), oxidative–antioxidant markers (MDA, GSH, CAT, TAS, TOS; spectrophotometry/ELISA), apoptosis and cell cycle (flow cytometry), and histopathology (H&E) were assessed. Relative to the control samples, both BA and TA significantly suppressed type-2 cytokines and TNF-α at protein and mRNA levels (all *p* < 0.01). Antioxidant responses increased with reductions in MDA and TOS and elevations in TAS and CAT (*p* < 0.01). MMP-9 decreased while TIMP-1 increased (*p* < 0.001), indicating improved ECM balance. At the cellular level, early apoptosis increased and G0/G1 accumulation suggested an antiproliferative effect. Histology showed a reduced inflammatory burden and oedema. In human nasal polyp tissue, we observed that BA attenuated inflammation and oxidative stress while supporting tissue homeostasis, demonstrating a multimodal therapeutic signal. Its combined effect profile with corticosteroids supports clinical evaluation of BA as an innovative adjuvant and a potential component of steroid-sparing strategies in CRSwNP management.

## Introduction

Chronic rhinosinusitis with nasal polyps (CRSwNP) is a severe inflammatory disorder predominantly driven by type 2 immunity and chronic oxidative stress [1, 2, 1–9]. Despite current treatment paradigms relying on intranasal corticosteroids and surgical intervention, postoperative recurrence remains common [1]. This clinical persistence underscores an urgent need for innovative strategies that concomitantly modulate tissue remodeling, oxidative stress, and inflammation.

In this context, boric acid (BA), a biologically active form of the naturally occurring element boron, has emerged as a promising candidate owing to its pleiotropic properties. Beyond its recognized anti-inflammatory and antioxidant capacities, BA has been implicated in fundamental cellular processes including maintenance of membrane integrity and regulation of the cell cycle [3]. Across experimental models, BA has been shown to neutralize reactive oxygen species, suppress proinflammatory mediators, and induce apoptosis in select cancer cell lines [4]. This pharmacological profile suggests potential utility in CRSwNP, where oxidative stress intersects with type 2 skewed immunity.

However, despite its promising profile in other biological systems, the specific impact of BA on the complex inflammatory and remodeling microenvironment of human nasal polyps remains largely unexplored. Specifically, it is unknown whether BA can disrupt the vicious cycle of oxidative stress and ECM degradation that often persists despite standard corticosteroid therapy. To address this knowledge gap, this study utilized a patient-matched ex vivo explant model, which preserves the native tissue architecture and immune milieu better than monolayer cell cultures. We hypothesized that BA would not only exert direct anti-inflammatory effects but also restore tissue homeostasis through a dual mechanism: modulating the oxidative balance and inhibiting pathological remodeling. Furthermore, we aimed to test whether BA could act as a ‘steroid-sparing’ adjuvant, potentially enhancing the efficacy of low-dose triamcinolone acetonide (TA) in CRSwNP management.

## Materials and Methods

### Study Design and Ethical Approval

This single-centre prospective cohort, incorporating an ex vivo, patient-matched randomized block experiment, was conducted at Kütahya Health Sciences University (KHSU). Ethics approval was granted by the Clinical Research Ethics Committee (2023-11/03). The study complied with the Declaration of Helsinki, and all participants provided written informed consent. Baseline disease severity was graded using the Lund–Kennedy endoscopy, Meltzer polyp, and Lund–Mackay CT scores [5–7].

### Study Population and Eligibility Criteria

**Fig. 1:**
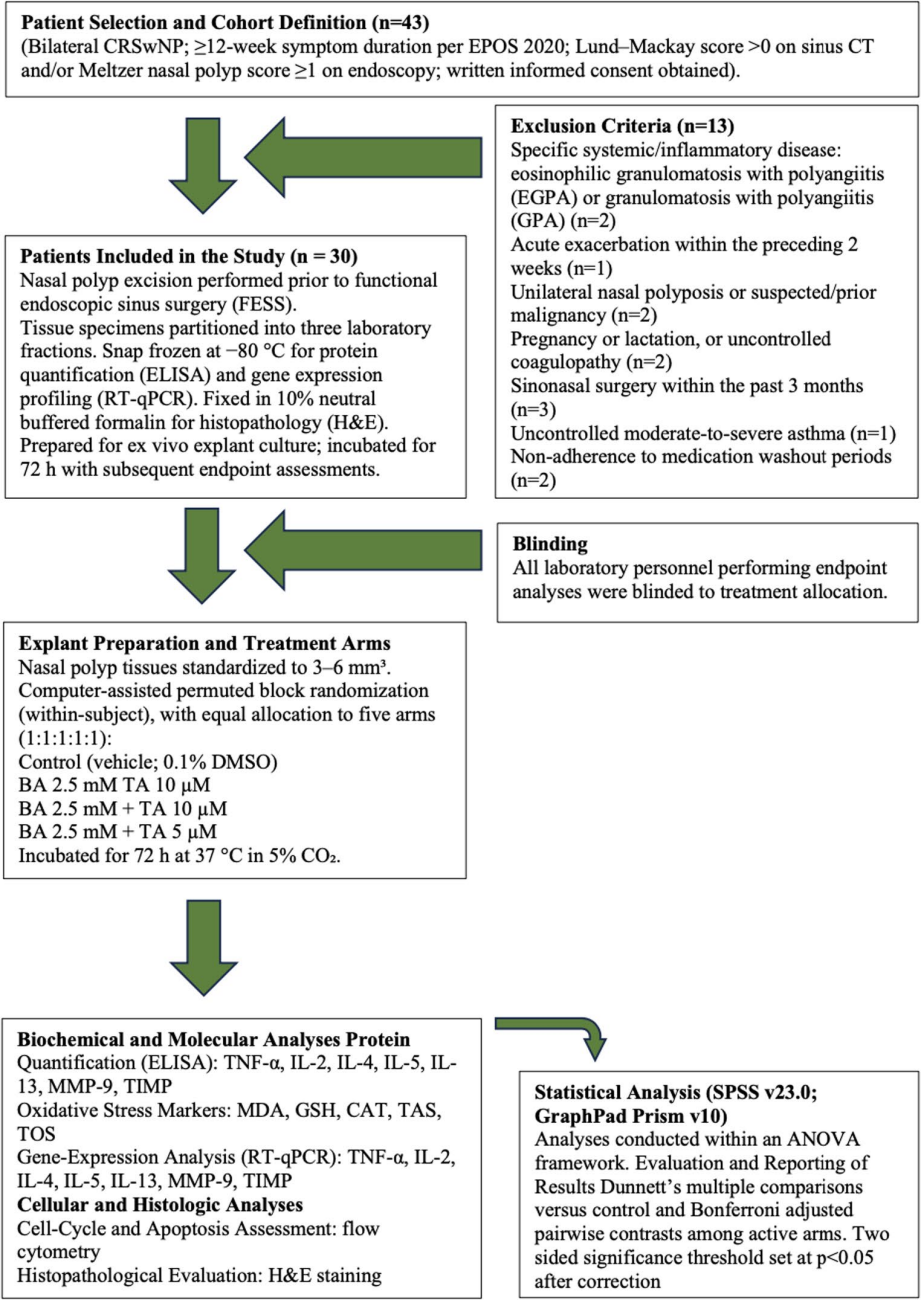
Flow diagram of patient enrollment, randomization, and analysis

Adults aged 18–75 years with CRSwNP, who were scheduled for functional endoscopic sinus surgery (FESS) were prospectively enrolled. A flow chart of patient enrollment, exclusion, preoperative washouts and analysis is presented in Fig. 1.

### Sample Size Determination

Assuming a two-sided hypothesis, medium correlation effect size (*r* = 0.33), 95% power (1 − β), and α = 0.05, a minimum of 30 patients was required [8].

### Tissue Procurement, Processing, *Ex Vivo* Culture, and Randomization

At the start of FESS, polyp tissue was aseptically excised from the middle meatus and transferred on ice in sterile PBS (pH 7.4) to the laboratory within 30 min. Specimens were partitioned for protein/qPCR (snap-frozen at − 80 °C), histology (10% neutral-buffered formalin), and ex vivo explant culture. Explants were rinsed, trimmed to ~ 3–6 mm³, placed mucosa-up in 6-well plates (DMEM + 10% FBS, penicillin 100 U/mL, streptomycin 100 µg/mL), and equilibrated 24 h at 37 °C, 5% CO₂. Each patient’s explants formed a randomization block. Computer-generated permuted-block randomization (block size 5) assigned wells (1:1:1:1:1) to vehicle (0.1% DMSO), BA 2.5 mM, TA 10 µM, BA 2.5 mM + TA 10 µM, or BA 2.5 mM + TA 5 µM. Allocation was concealed with identical opaque coded tubes; operators and assay staff were blinded until database lock. All wells contained 0.1% DMSO and were incubated for 72 h.

### Dose Justification

The BA 2.5 mM was selected from a 48-h MTT cytotoxicity screen in L929 fibroblasts (IC₅₀ = 47.92 mM), which preserved ≥ 80% viability. BA 2.5 mM corresponds to approximately 155 mg/L (≈ 0.015% w/v) and was selected as a conservative, tissue-compatible exposure when contrasted with boric acid concentrations historically reported in topical formulations, which are typically in the percent range [9]. In the absence of validated pharmacokinetic data defining achievable boric acid levels in human nasal mucosa, we deliberately opted for a concentration substantially below those used in topical preparations and below cytotoxicity thresholds identified in preliminary screening assays, to minimize nonspecific tissue injury and to ensure interpretability of explant outcomes as pharmacodynamic effects rather than toxicity-driven artefacts [10]. TA was applied at 5 or 10 µM, consistent with therapeutic concentrations reported in the literature [11, 12]. TA 5–10 µM corresponds to ~ 2.17–4.35 µg/mL. Intranasal sprays deliver 55 µg per actuation. However, effective mucosal concentrations vary with deposition and dilution in nasal surface liquid. Therefore, these explant doses were selected as conservative, mechanistically informative exposures supported by prior experimental studies, rather than as direct surrogates of in vivo luminal levels.

### Quantitative Real-Time PCR (RT-qPCR) for Gene Expression

After 72 h, total RNA was extracted from explants (NucleoGene) and assessed by NanoDrop (A260/A280, A260/A230). cDNA was synthesized with a reverse-transcription kit (NucleoGene). TNF-α, IL-2, IL-4, IL-5, IL-13, MMP-9, and TIMP-1 mRNAs were quantified by RT-qPCR (ABI StepOne™ Plus; SYBR Green) using β-actin as the reference gene. Relative expression was calculated by the 2^−ΔΔ^Cq method versus vehicle. All the reactions included three biological and two technical replicates.

## Measurement of Oxidative Stress Markers

Tissues were bead-homogenized on ice; supernatants were collected after centrifugation (10,000 × g, 15 min, 4 °C). MDA was measured by TBARS; GSH by DTNB (Ellman’s reagent); CAT by H₂O₂ decomposition at 240 nm; TAS/TOS with commercial kits (Elabscience, USA) per manufacturer instructions. Results were normalized to total protein (Bradford) and reported per mg protein.

### Enzyme Linked Immunosorbent Assay (ELISA)

IL-4, IL-5, IL-13, TNF-α, MMP-9, and TIMP-1 in tissue homogenates were quantified by ELISA (Reed Biotech, China) per manufacturer instructions. Standards/samples were run in duplicate; absorbance was read at 450 nm (Thermo Scientific Multiskan). Concentrations were interpolated from standard curves, normalized to total protein.

### Flow Cytometry for Cell Cycle and Apoptosis Analysis

Post-treatment explants were mechanically dissociated to single cell suspensions, filtered (50 μm), and stained for apoptosis with Annexin V–FITC/7-AAD (Elabscience) and for cell cycle with DRAQ5 (Cell Signaling Technology). Data were acquired on a CytoFLEX (Beckman Coulter, CA, USA). Debris/aggregates were excluded by FSC/SSC gating. A minimum of 5,000 events was collected per sample.

### Histopathology Assessment

After 72 h of culture, tissues were fixed in 10% neutral buffered formalin for 24 h, processed, paraffin-embedded, and sectioned at 5 μm using a rotary microtome before being stained with hematoxylin and eosin (H&E) for examination under a Zeiss Axiolab 5 microscope by an experienced investigator blinded to group allocation. Semi-quantitative histological evaluation was performed by scoring three specific parameters; inflammatory cell infiltration, stromal edema, and vascular congestion on a scale from 0 (none/absent) to 3 (severe/diffuse) in five randomly selected areas per group (presented as Mean ± SD), based on criteria adapted from Gibson-Corley et al. [13] and Mygind and Lildholdt [14].

### Statistical Analysis

Analyses were performed using SPSS (v23.0) and GraphPad Prism (v10). Data are presented as mean ± SD or median (IQR), as appropriate. Between-group differences were assessed using one-way ANOVA. When the omnibus test was significant, Dunnett’s post hoc test was used for comparisons versus the vehicle control. To control the family-wise error rate across the full set of pairwise comparisons among the active treatment arms, Bonferroni correction was applied and adjusted p-values are reported. All tests were two-sided, and *p* < 0.05 was considered statistically significant.

## Results

### Baseline Demographic and Clinical Characteristics

Evaluations were made of 30 patients (median age, 44 years [18–66]); 66.7% male). The median Charlson Comorbidity Index value was 1 (0–5), and median Lund-Mackay CT scores were 8 (left) and 9 (right). Additional demographic and clinical variables are summarized in Table 1.

**Table 1:** Demographic and clinical characteristics of patients with CRSwNP

Characteristic	Value
Gender	Female: 10 (33.3%)
	Male: 20 (66.7%)
Age,(years) median (min–max)	44 (18–66)
Charlson Comorbidity Index, median (min–max)	1 (0–5)
Meltzer polyp score, median (min–max)	3 (1–4)
Lund–Kennedy endoscopy score, median (min–max)	5 (2–7)
Lund–Mackay CT score - LEFT, median (min–max)	8 (4–12)
Lund–Mackay CT score - RIGHT, median (min–max)	9 (4–12)

### Cytokines and ECM Markers

**Fig. 2:**
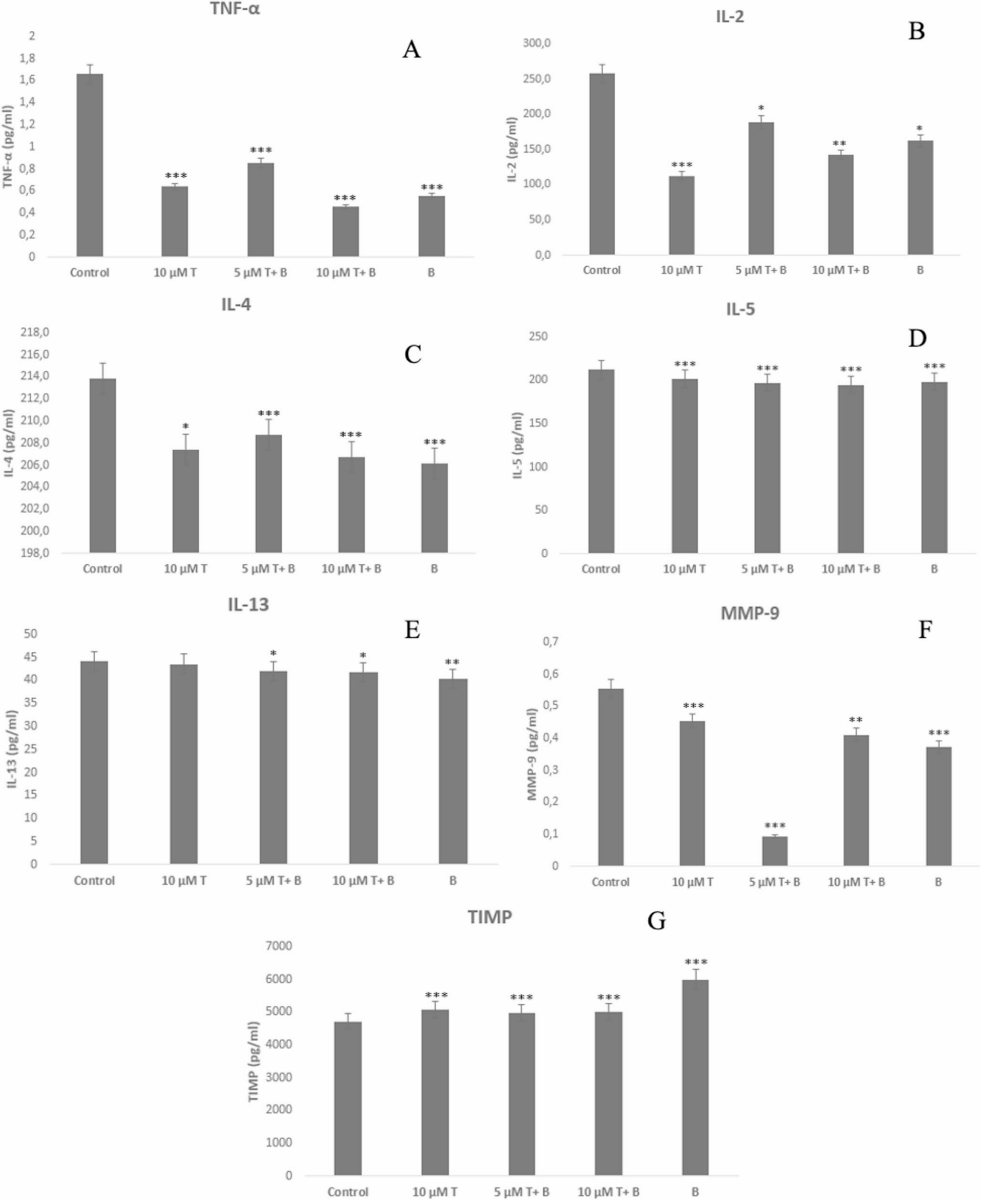
Cytokines and ECM markers at 72 h (pg/mL). Panels: (**A**) TNF-α, (**B**) IL-2, (**C**) IL-4, (**D**) IL-5, (**E**) IL-13, (**F**) MMP-9, (**G**) TIMP-1. Groups: Control, TA 10 μM, TA 5 μM + BA 2.5 mM, TA 10 μM + BA 2.5 mM, BA 2.5 mM. Oxidative stress indices at 72 h. Panels: (H) MDA, (I) GSH, (J) CAT, (K) TAS, (L) TOS. Data are presented as mean ± standard deviation (SD) values. TA, triamcinolone acetonide; BA, boric acid. *p < 0.05, **p < 0.01, ***p < 0.001 ; Dunnett-adjusted (vs control). Exact adjusted p values are provided below. Exact Dunnett-adjusted p values (vs control): (A) TNF-α: TA10 p<0.0001; TA5+BA p<0.0001; TA10+BA p<0.0001; BA p<0.0001. (B) IL-2: TA10 p=0.0008; TA5+BA p=0.2126; TA10+BA p=0.0110; BA p=0.0458. (C) IL-4: TA10 p=1.22×10⁻¹⁵; TA5+BA p=2.16×10⁻¹¹; TA10+BA p<0.0001; BA p<0.0001. (D) IL-5: TA10 p<0.0001; TA5+BA p<0.0001; TA10+BA p<0.0001; BA p<0.0001. (E) IL-13: TA10 p=0.0263; TA5+BA p=4.30×10⁻⁶; TA10+BA p=9.05×10⁻⁷; BA p=1.67×10⁻¹⁵. (F) MMP-9: TA10 p=2.52×10⁻⁸; TA5+BA p<0.0001; TA10+BA p=3.77×10⁻¹⁵; BA p<0.0001. (G) TIMP-1: TA10 p<0.0001; TA5+BA p<0.0001; TA10+BA p<0.0001; BA p<0.0001. (H) MDA: TA10 p<0.0001; TA5+BA p<0.0001; TA10+BA p<0.0001; BA p<0.0001. (I) GSH: TA10 p=0.3362; TA5+BA p=0.7544; TA10+BA p=1.67×10⁻⁶; BA p=4.41×10⁻¹⁰. (J) CAT: TA10 p<0.0001; TA5+BA p<0.0001; TA10+BA p=0.0005; BA p<0.0001. (K) TAS: TA10 p<0.0001; TA5+BA p=2.24×10⁻⁵; TA10+BA p<0.0001; BA p<0.0001. (L) TOS: TA10 p=0.0002; TA5+BA p=1.24×10⁻¹⁴; TA10+BA p<0.0001; BA p<0.0001

Across arms, pro-inflammatory cytokines fell versus vehicle: TNF-α, IL-4, IL-5, and IL-13 decreased in all groups (all adjusted *p* < 0.05), while IL-2 decreased with TA 10 µM, BA 2.5 mM, and BA + TA 10 µM (BA + TA 5 µM: ns). Among active arms, the most pronounced effects were: TNF-α-BA + TA 10 µM lowest (also < TA 10 µM and < BA + TA 5 µM), with BA 2.5 mM ≈ BA + TA 10 µM; IL-5-BA + TA 10 µM < TA 10 µM and < BA 2.5 mM; IL-13-BA 2.5 mM lowest (below TA 10 µM and both combinations). For ECM remodeling, MMP-9 decreased across all groups (lowest with BA + TA 5 µM, below all others), while TIMP-1 increased in all groups (highest with BA 2.5 mM), and consistent with suppression of inflammatory/oxidative drivers pathological tissue remodeling was significantly halted, with MMP-9 downregulated in every arm. See Table 2; Fig. 2 for values and post-hoc tests.

**Table 2:** Effects of TA (5–10 µM) and/or BA (2.5 mM) on inflammatory cytokines (IL-2, IL-4, IL-5, IL-13, TNF-α), MMP-9/TIMP-1, and oxidative indices (MDA, GSH, CAT, TAS, TOS)

Marker (unit)	Control	T 10 µM	T 5 µM + BA 2.5 mM	T 10 µM + BA 2.5 mM	BA 2.5 mM	*p*-values (ANOVA; Dunnett vs. control) + mean difference (Δ) vs. control (95% CI)	Selected pairwise *p*-values (Bonferroni; active arms)
TNF-α (pg/mL)	1.66 ± 0.50	0.638 ± 0.22	0.850 ± 0.12	0.453 ± 0.28	0.554 ± 0.10	**ANOVA: p=7.28×10**^**-38**^ **Dunnett vs control (adjusted p):** **T 10 μM:Δ=-1.022 (-1.224 to -0.8204);** ***p*<0.0001** **T 5 μM+BA:Δ=-0.8100 (-1.001 to -0.6189);** ***p*<0.0001** **T 10 μM+BA: Δ=-1.207** **(-1.418 to -0.9963);** ***p*<0.0001** **BA 2.5 mM: Δ=-1.106 (-1.296 to -0.9162);** ***p*<0.0001**	**Bonferroni pairwise (selected active arms):** **T 10 μM vs T 5 μM+BA: *p*=0.026** **T 5 μM+BA vs T 10 μM+BA:** ***p* =1.4×10-6** **T 5 μM+BA vs BA 2.5 mM:*p*=0.0005**
IL-2 (pg/mL)	257.7 ± 216.6	112.4 ± 87.8	188.2 ± 158.3	142.4 ± 140.3	162.0 ± 101.2	**ANOVA: p=0.0033Dunnett vs control (adjusted p):** **T 10 μM: Δ=-145.3 (-231.7 to -58.94);*p*=0.0008** **T 5 μM+BA:Δ=-69.50 (-167.7 to 28.74);*p*=0.2126** **T 10 μM+BA: Δ=-115.3 (-210.0 to -20.65);*p*=0.011** **BA 2.5 mM: Δ=-95.70 (-183.8 to -7.555);** ***p*=0.0458**	**Bonferroni pairwise (selected active arms):** **T 10 µM vs. T 5 µM + BA:** ***p* = 0.2957** **T 10 µM vs. T 10 µM + BA:** ***p* = 1.000** **T 10 µM vs. BA 2.5 mM:** ***p* = 1.000** **T 5 µM + BA vs. T 10 µM + BA:** ***p* = 1.000** **T 5 µM + BA vs. BA 2.5 mM: *p* = 1.000** **T 10 µM + BA vs. BA 2.5 mM:*p* = 1.000**
IL-4 (pg/mL)	**213.9 ± 3.1**	**207.4 ± 3.2**	**208.7 ± 1.6**	**206.7 ± 1.5**	**206.1 ± 3.6**	**ANOVA: p=3.02×10–22** **Dunnett vs control (adjusted p):** **T 10 μM: Δ=−6.500> (−8.128 to −4.872);** ***p*=1.22×10–15 ** **T 5 μM+BA: Δ=−5.200 (−6.484 to −3.916);*p*=2.16×10–11 ** **T 10 μM+BA: Δ=−7.200 (−8.469 to −5.931);** ***p*<0.0001 ** **BA 2.5 mM: Δ=−7.800 (−9.537 to −6.063);** ***p*<0.0001**	**Bonferroni pairwise (selected active arms): ** **T 10 μM vs T 5 μM+BA:*p*=0.4108 ** **T 10 μM vs T 10 μM+BA:*p*=1.000 ** **T 10 μM vs BA 2.5 mM:*p*=0.4108 ** **T 5 μM+BA vs T 10 μM+BA:** ***p*=0.0325 ** **T 5 μM+BA vs BA 2.5 mM:*p*=0.002 ** **T 10 μM+BA vs BA 2.5 mM:*p*=1.000**
IL-5 (pg/mL)	**212 ± 3.27**	**202 ± 3.60**	**195 ± 3.61**	**197 ± 3.80**	**199 ± 3.61**	**ANOVA:** ***p*=1.34×10^-41** **Dunnett vs control (adjusted p):** **T 10 μM: Δ=-10.00 (-11.78 to -8.222); *p***<**0.0001 ** **T 5 μM+BA: Δ=-17.00 (-18.78 to -15.22);** ***p*<0.0001 ** **T 10 μM+BA: Δ=-15.00 (-16.83 to -13.17);p<0.0001 ** **BA 2.5 mM: Δ=-13.00 (-14.78 to -11.22); *p*<0.0001**	**Bonferroni pairwise (selected active arms):** **T 10 μM vs T 5 μM+BA:*p*=2.41×10-11 ** **T 10 μM vs T 10 μM+BA:** ***p*=1.55×10-6 ** **T 10 μM vs BA 2.5 mM:** ***p*=0.0088 ** **T 5 μM+BA vs T 10 μM+BA:** ***p*=0.1934 ** **T 5 μM+BA vs BA 2.5 mM:** ***p*=0.0002 ** **T 10 μM+BA vs BA 2.5 mM:** ***p*=0.1934**
IL-13 (pg/mL)	**44.14 ± 1.41**	**43.0 ± 1.58**	**42.0 ± 1.58**	**41.86 ± 1.58**	**40.42 ± 1.94**	**ANOVA: p=4.29×10^-14 ** **Dunnett vs control (adjusted p):** **T 10 μM: Δ=-1.140 (-1.914 to -0.3659);** ***p*=0.0263 ** **T 5 μM+BA: Δ=-2.140 (-2.914 to -1.366);** ***p*=4.30×10-6 ** **T 10 μM+BA: Δ=-2.280 (-3.054 to -1.506);*p*=9.05×10-7 ** **BA 2.5 mM: Δ=-3.720 (-4.598 to -2.842);*p*=1.67×10-15**	**Bonferroni pairwise (selected active arms):** **T 10 μM vs T 5 μM+BA:** ***p*=0.1117** **T 10 μM vs T 10 μM+BA:** ***p*=0.0448** **T 10 μM vs BA 2.5 mM:** ***p*=4.50×10-8** **T 5 μM+BA vs T 10 μM+BA:** ***p*=1.000** **T 5 μM+BA vs BA 2.5 mM:** ***p*=0.0015** **T 10 μM+BA vs BA 2.5 mM:** ***p*=0.0048**
MMP-9 (pg/mL)	**0.555 ± 0.072**	**0.454 ± 0.072**	**0.094 ± 0.013**	**0.410 ± 0.071**	**0.373 ± 0.072**	**ANOVA: p=7.82×10^-60** **Dunnett vs control (adjusted p):** **T 10 μM: Δ=-0.1010 (-0.1382 to -0.06379);** ***p*=2.52×10-8** **T 5 μM+BA: Δ=-0.4610 (-0.4882 to -0.4338);*p*<0.0001** **T 10 μM+BA: Δ=-0.1450 (-0.1820 to -0.1080);*p*=3.77×10^-15** **BA 2.5 mM: Δ=-0.1820 (-0.2192 to -0.1448);*p*<0.0001**	**Bonferroni pairwise (selected active arms):** **T 10 μM vs T 5 μM+BA:** ***p*=1.80×10-46** **T 10 μM vs T 10 μM+BA:** ***p*=0.0545** **T 10 μM vs BA 2.5 mM:** ***p*=1.74×10-5** **T 5 μM+BA vs T 10 μM+BA:** ***p*=2.17×10-4** **T 5 μM+BA vs BA 2.5 mM:** ***p*=5.50×10-35** **T 10 μM+BA vs BA 2.5 mM:** ***p*=0.1662**
TIMP-1 (pg/mL)	**4715 ± 22.36**	**5073 ± 72.11**	**4973 ± 70.11**	**5004 ± 71.06**	**6000 ± 25.12**	**ANOVA: p=4.08×10^-129** **Dunnett vs control (adjusted p):** **T 10 μM: Δ=358.0 (330.0 to 386.0);** ***p*<0.0001** **T 5 μM+BA: Δ=258.0 (230.7 to 285.3);*p*<0.0001** **T 10 μM+BA: Δ=289.0 (261.4 to 316.6);*p*<0.0001** **BA 2.5 mM: Δ=1285.0 (1272.7 to 1297.3);*p*<0.0001**	**Bonferroni pairwise (selected active arms):** **T 10 μM vs T 5 μM+BA:** ***p*=1.66×10-9** **T 10 μM vs T 10 μM+BA:** ***p*=3.90×10-5** **T 10 μM vs BA 2.5 mM:** ***p*=2.39×10-106** **T 5 μM+BA vs T 10 μM+BA:** ***p*=0.2231** **T 5 μM+BA vs BA 2.5 mM:** ***p*=1.36×10-112** **T 10 μM+BA vs BA 2.5 mM:** ***p*=1.01×10-110**
MDA (nmol/g)	**0.0558 ± 0.0029**	**0.0395 ± 0.0026**	**0.0400 ± 0.0025**	**0.0397 ± 0.0036**	**0.0379 ± 0.0016**	**ANOVA:** ***p*=4.79×10^-61 ** **Dunnett vs control (adjusted p):** **T 10 μM: Δ=-0.01630 (-0.01772 to -0.01488);** ***p*<0.0001 ** **T 5 μM+BA: Δ=-0.01580 (-0.01720 to -0.01440);*p*<0.0001 ** **T 10 μM+BA:Δ=-0.01610 (-0.01779 to -0.01441);** ***p*<0.0001** **BA 2.5 mM: Δ=-0.01790 (-0.01912 to -0.01668);** ***p*<0.0001**	**Bonferroni pairwise (selected active arms):** ** T 10 μM vs T 5 μM+BA:** ***p*=1.000 ** ** T 10 μM vs T 10 μM+BA:** ***p*=1.000 ** ** T 10 μM vs BA 2.5 mM:** ***p*=0.1445 ** ** T 5 μM+BA vs T 10 μM+BA:** ***p*=1.000 ** ** T 5 μM+BA vs BA 2.5 mM:** ***p*=0.0195 ** ** T 10 μM+BA vs BA 2.5 mM:** ***p*=0.068**
GSH (nmol/g)	**0.0324 ± 0.0010**	**0.0329 ± 0.0014**	**0.0327 ± 0.0021**	**0.0341 ± 0.0004**	**0.0346 ± 0.0004**	**ANOVA: p=6.50×10^-12** **Dunnett vs control (adjusted p):** **T 10 μM: Δ=0.00050 (-0.00013 to 0.00113);** ***p*=0.3362** **T 5 μM+BA: Δ=0.00030 (-0.00056 to 0.00116);** ***p*=0.7544** **T 10 μM+BA: Δ=0.00170 (0.00130 to 0.00210);** ***p*=1.67×10-6** **BA 2.5 mM: Δ=0.00220 (0.00180 to 0.00260);** ***p*=4.41×10-10**	**Bonferroni pairwise (selected active arms):** **T 10 μM vs T 5 μM+BA:** ***p*=1.000** **T 10 μM vs T 10 μM+BA:** ***p*=0.0015** **T 10 μM vs BA 2.5 mM:** ***p*=2.43×10-6** **T 5 μM+BA vs T 10 μM+BA:** ***p*=0.0001** **T 5 μM+BA vs BA 2.5 mM:** ***p*=1.25×10-7** **T 10 μM+BA vs BA 2.5 mM:** ***p*=0.7235**
CAT (U/mg)	**1.02 ± 0.03**	**1.14 ± 0.02**	**1.26 ± 0.07**	**1.07 ± 0.03**	**1.29 ± 0.07**	**ANOVA: p=6.22×10^-54** **Dunnett vs control (adjusted p):** **T 10 μM: Δ=0.1200 (0.1068 to 0.1332);** ***p*<0.0001** **T 5 μM+BA: Δ=0.2400 (0.2119 to 0.2681);** ***p*<0.0001** **T 10 μM+BA: Δ=0.05000 (0.03449 to 0.06551);** ***p*=0.0005** **BA 2.5 mM: Δ=0.2700 (0.2419 to 0.2981);** ***p*<0.0001**	**Bonferroni pairwise (selected active arms):** **T 10 μM vs T 5 μM+BA: *p*=3.97×10-16** **T 10 μM vs T 10 μM+BA:** ***p*=8.54×10-7** **T 10 μM vs BA 2.5 mM: *p*=2.53×10-22** **T 5 μM+BA vs T 10 μM+BA: *p*=1.43×10-30** **T 5 μM+BA vs BA 2.5 mM: *p*=0.1141** **T 10 μM+BA vs BA 2.5 mM: *p*=1.58×10-36**
TAS (mmol Trolox Eqv/L)	**0.17 ± 0.04**	**0.38 ± 0.03**	**0.22 ± 0.05**	**0.33 ± 0.03**	**0.39 ± 0.05**	**ANOVA: p=4.31×10^-54** **Dunnett vs control (adjusted p):** **T 10 μM: Δ=0.2100 (0.1917 to 0.2283);** ***p*<0.0001** **T 5 μM+BA: Δ=0.05000 (0.02657 to 0.07343);** ***p*=2.24×10-5** **T 10 μM+BA: Δ=0.1600 (0.1417 to 0.1783);*p*<0.0001** **BA 2.5 mM: Δ=0.2200 (0.1966 to 0.2434); *p*<0.0001**	**Bonferroni pairwise (selected active arms):** **T 10 μM vs T 5 μM+BA:*p*=8.05×10-31** **T 10 μM vs T 10 μM+BA:*p*=3.24×10-5** **T 10 μM vs BA 2.5 mM:** ***p*=1.000** **T 5 μM+BA vs T 10 μM+BA:*p*=1.77×10-18** **T 5 μM+BA vs BA 2.5 mM:** ***p*=3.20×10-33** **T 10 μM+BA vs BA 2.5 mM:** ***p*=4.49×10-7**
TOS (µmol H₂O₂ Eqv/L)	**2.3 ± 0.28**	**2.05 ± 0.30**	**1.78 ± 0.22**	**1.40 ± 0.15**	**1.60 ± 0.16**	**ANOVA: p=1.62×10^-33** ** Dunnett vs control (adjusted p):** **T 10 μM: Δ=-0.2500 (-0.4000 to -0.1000);** ***p*=0.0002** **T 5 μM+BA: Δ=-0.5200 (-0.6503 to -0.3897);*p*=1.24×10-14** **T 10 μM+BA: Δ=-0.9000 (-1.017 to -0.7831);*p*<0.0001** ** BA 2.5 mM: Δ=-0.7000 (-0.8185 to -0.5815);*p*<0.0001**	**Bonferroni pairwise (selected active arms):** **T 10 μM vs T 5 μM+BA:** ***p*=6.95×10-5** **T 10 μM vs T 10 μM+BA:*p*=6.71×10-20** **T 10 μM vs BA 2.5 mM:** ***p*=2.37×10-11** **T 5 μM+BA vs T 10 μM+BA:** ***p*=1.25×10-8** **T 5 μM+BA vs BA 2.5 mM: *p*=0.0174** **T 10 μM+BA vs BA 2.5 mM: *p*=0.0059**

Data are presented as mean ± SD; *n* = 30 per group. Overall differences among groups were assessed using one-way ANOVA. Comparisons versus the control group were performed using Dunnett’s multiple-comparisons test (multiplicity-adjusted p values), with effect estimates reported as mean differences (Δ) and corresponding 95% confidence intervals (CIs). Selected active-arm pairwise comparisons were evaluated using Bonferroni-adjusted p values. All tests were two-sided. Very small p values are reported in scientific notation or as *p* < 0.0001, consistent with GraphPad Prism output. Abbreviations: *BA* boric acid, *CAT* catalase, *CI* confidence interval, *GSH* reduced glutathione, *IL* interleukin, *MDA* malondialdehyde, *MMP-9* matrix metalloproteinase-9, *TA* triamcinolone acetonide, *TAS* total antioxidant status, *TIMP-1* tissue inhibitor of metalloproteinases-1, *TNF-α* tumor necrosis factor-alpha, *TOS* total oxidant status

### Oxidative Stress Indices

MDA decreased in all groups (lowest with BA 2.5 mM). GSH showed nominal rises with BA 2.5 mM and BA + TA 10 µM but was not significant after correction. CAT increased in all groups (BA 2.5 mM and BA + TA 5 µM > TA 10 µM and BA + TA 10 µM). TAS increased in all groups (BA 2.5 mM and TA 10 µM > both combinations). TOS decreased in all groups (lowest with BA + TA 10 µM). **See** Table 2**and** Fig. 2.

Notably, the reduction in oxidative stress markers (MDA, TOS) and the concomitant rise in antioxidant capacity (TAS, CAT) closely paralleled the downregulation of pro-inflammatory cytokines, suggesting a linked redox-inflammatory modulation by BA and TA.

### Gene Expression (RT-qPCR)

**Fig. 3:**
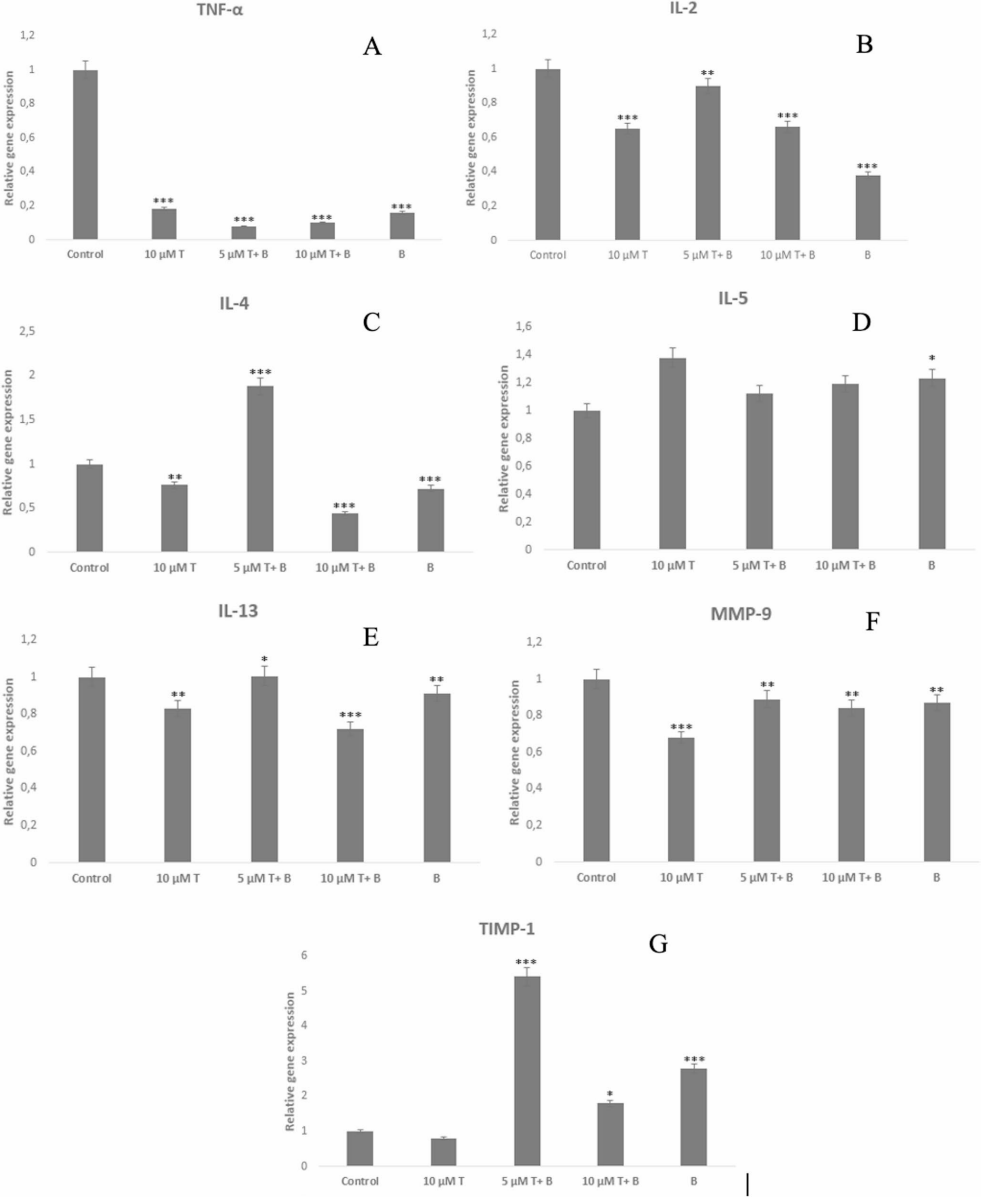
Gene expression levels at 72 h after TA 10 μM, BA 2.5 mM, TA 10 μM + BA 2.5 mM, and TA 5 μM + BA 2.5 mM. Panels: (**A**) TNF-α, (**B**) IL-2, (**C**) IL-4, (**D**) IL-5, (**E**) IL-13, (**F**) MMP-9, (**G**) TIMP-1. Groups: Control, TA 10 μM, TA 5 μM + BA 2.5 mM, TA 10 μM + BA 2.5 mM, BA 2.5 mM. Data are presented as mean ± SD. One-way ANOVA across groups; Dunnett’s multiple-comparisons test versus control (two-sided, multiplicity-adjusted). *p<0.05, **p<0.01, ***p<0.001 versus control. Exact Dunnett-adjusted p values (vs control): (A) TNF-α: TA10 p<0.001; TA5+BA p<0.001; TA10+BA p<0.001; BA p<0.001. (B) IL-2: TA10 p<0.001; TA5+BA p=0.007; TA10+BA p<0.001; BA p<0.001. (C) IL-4: TA10 p=0.001; TA5+BA p<0.001; TA10+BA p<0.001; BA p<0.001. (D) IL-5: TA10 p=0.079; TA5+BA p=0.327; TA10+BA p=0.248; BA p=0.032. (E) IL-13: TA10 p=0.002; TA5+BA p=0.024; TA10+BA p<0.001; BA p=0.008. (F) MMP-9: TA10 p<0.001; TA5+BA p=0.005; TA10+BA p=0.002; BA p=0.003. (G) TIMP-1: TA10 p=0.105; TA5+BA p<0.001; TA10+BA p=0.010; BA p<0.001.

We observed broad anti-inflammatory transcriptional shifts across treatment arms: TNF-α was strongly suppressed in all groups (0.08–0.18-fold; greatest with BA + TA 5 µM), IL-2 decreased in all (maximal with BA 2.5 mM, 0.38-fold), and IL-4 fell with TA 10 µM, BA 2.5 mM, and BA + TA 10 µM but rose with BA + TA 5 µM (1.88-fold). Consistent with the suppression of these inflammatory and oxidative drivers, pathological tissue remodeling was also significantly halted. IL-5 showed no meaningful overall change (small increase with BA 2.5 mM), IL-13 decreased in all but BA + TA 5 µM (≈ control), MMP-9 was downregulated in every arm, and TIMP-1 was induced by all BA-containing regimens (maximal with BA + TA 5 µM, 5.41-fold) while TA 10 µM was ≈ control. Statistical details (one-way ANOVA with Bonferroni) are shown in Fig. 3, with fold-change/log₂FC/^ΔΔ^Ct summarized in Table 3.

**Table 3:** The effects of TA and BA on gene expression in CRSwNP explants by RT-qPCR: fold-change, log₂FC, ΔΔCt and adjusted *p* values vs. control

Gene	Group	Fold-change (vs. Control)	log2FC	ΔΔCt	Direction	Dunnett vs. control (adjusted *p*)
TNF-α	T 10 µM	0.18	-2.4739	2.4739	↓ (down)	*p* < 0.001
TNF-α	T 10 µM + BAA 2.5 mM	0.18	-2.4739	2.4739	↓ (down)	*p* < 0.001
TNF-α	T 5 µM + BAA 2.5 mM	0.08	-3.6439	3.6439	↓ (down)	*p* < 0.001
TNF-α	BA 2.5 mM	0.16	-2.6439	2.6439	↓ (down)	*p* < 0.001
IL-2	T 10 µM	0.65	-0.6215	0.6215	↓ (down)	*p* < 0.001
IL-2	T 10 µM + BAA 2.5 mM	0.66	-0.5995	0.5995	↓ (down)	*p* < 0.001
IL-2	BA 2.5 mM	0.38	-1.3959	1.3959	↓ (down)	*p* < 0.001
IL-2	T 5 µM + BAA 2.5 mM	0.9	-0.1520	0.1520	↓ (down)	*p* = 0.007
IL-4	T 10 µM	0.76	-0.3959	0.3959	↓ (down)	*p* = 0.001
IL-4	T 10 µM + BAA 2.5 mM	0.44	-1.1844	1.1844	↓ (down)	*p* < 0.001
IL-4	BA 2.5 mM	0.72	-0.4739	0.4739	↓ (down)	*p* < 0.001
IL-4	T 5 µM + BAA 2.5 mM	1.88	0.9107	-0.9107	↑ (up)	*p* < 0.001
IL-5	BA 2.5 mM	1.23	0.2987	-0.2987	↑ (up)	*p* = 0.032
IL-5	T 10 µM	1.38	0.4647	-0.4647	↑ (up)	*p* = 0.079
IL-5	T 10 µM + BAA 2.5 mM	1.19	0.2510	-0.2510	↑ (up)	*p* = 0.248
IL-5	T 5 µM + BAA 2.5 mM	1.12	0.1635	-0.1635	↑ (up)	*p* = 0.327
IL-13	T 10 µM	0.83	-0.2688	0.2688	↓ (down)	*p* = 0.002
IL-13	T 10 µM + BAA 2.5 mM	0.72	-0.4739	0.4739	↓ (down)	*p* < 0.001
IL-13	BA 2.5 mM	0.91	-0.1361	0.1361	↓ (down)	*p* = 0.008
IL-13	T 5 µM + BAA 2.5 mM	1.005	0.0072	-0.0072	↑ (up)	*p* = 0.024
MMP-9	T 10 µM	0.68	-0.5564	0.5564	↓ (down)	*p* < 0.001
MMP-9	T 10 µM + BAA 2.5 mM	0.84	-0.2515	0.2515	↓ (down)	*p* = 0.002
MMP-9	T 5 µM + BAA 2.5 mM	0.89	-0.1681	0.1681	↓ (down)	*p* = 0.005
MMP-9	BA 2.5 mM	0.87	-0.2009	0.2009	↓ (down)	*p* = 0.003
TIMP-1	T 10 µM	0.79	-0.3401	0.3401	↓ (down)	*p* = 0.105
TIMP-1	T 10 µM + BAA 2.5 mM	1.8	0.8480	-0.8480	↑ (up)	*p* = 0.010
TIMP-1	T 5 µM + BAA 2.5 mM	5.41	2.4356	-2.4356	↑ (up)	*p* < 0.001
TIMP-1	BA 2.5 mM	2.79	1.4803	-1.4803	↑ (up)	*p* < 0.001

*Data are presented as fold-change relative to the control group.log₂FC was calculated as log₂(fold-change)*,* and ΔΔCt as − log₂FC.* *P values are two-sided and adjusted for multiple comparisons versus control (Dunnett)*. *Values shown as* *p < 0.001 correspond to p values reported as 0.000 in the original exported output.*

### Apoptosis (Flow Cytometry)

**Fig. 4:**
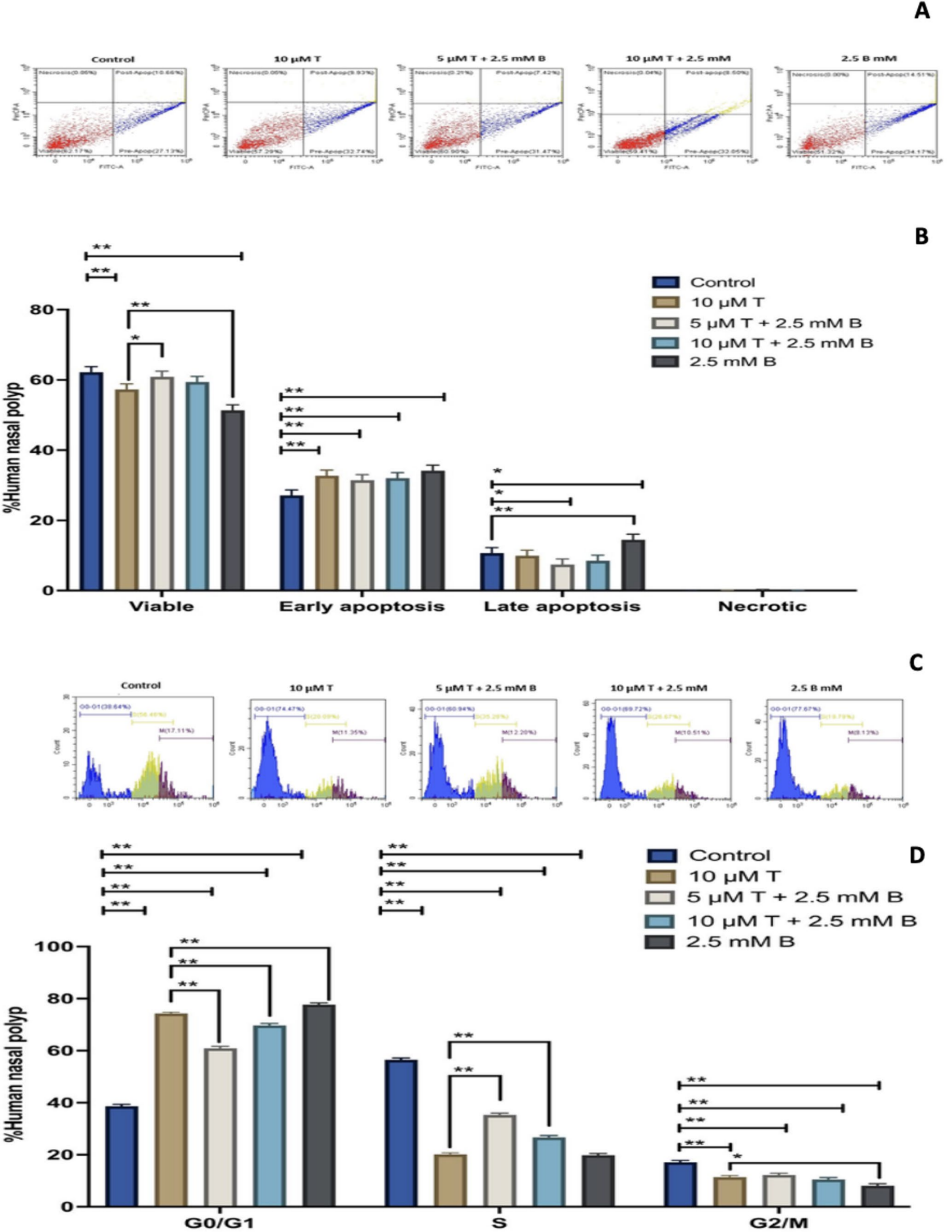
Cell-death profile at 72 h by flow cytometry. Data are presented as mean ± SD (*n*=5 per group). Overall group differences were assessed using one-way ANOVA; comparisons versus control were performed with Dunnett’s multiple-comparisons test (adjusted p values). Exact Dunnett-adjusted p values (vs control) were as follows: Viable-TA 10 μM p=3.97×10⁻⁴, TA 5 μM+BA 2.5 mM p=0.534, TA 10 μM+BA 2.5 mM p=0.0399, BA 2.5 mM p=6.72×10⁻¹⁰; Early apoptosis-TA 10 μM p=7.45×10⁻⁵, TA 5 μM+BA p=0.0012, TA 10 μM+BA p=3.16×10⁻⁴, BA p=2.31×10⁻⁶; Late apoptosis-TA 10 μM p=0.876, TA 5 μM+BA p=0.0141, TA 10 μM+BA p=0.131, BA p=0.0036; Necrosis-TA 10 μM p=1.000, TA 5 μM+BA p=0.0074, TA 10 μM+BA p=0.998, BA p=0.647; G0/G1-TA 10 μM p=5.12×10⁻¹⁹, TA 5 μM+BA p=5.36×10⁻¹⁵, TA 10 μM+BA p=8.39×10⁻¹⁸, BA p=9.47×10⁻²⁰; S phase-TA 10 μM p=3.81×10⁻¹⁹, TA 5 μM+BA p=1.44×10⁻¹⁴, TA 10 μM+BA p=1.93×10⁻¹⁷, BA p=3.24×10⁻¹⁹; G2/M-TA 10 μM p=4.42×10⁻⁵, TA 5 μM+BA p=2.99×10⁻⁴, TA 10 μM+BA p=8.00×10⁻⁶, BA p=1.33×10⁻⁸. Between-treatment comparisons (active arms) displayed in the figure were evaluated using Bonferroni adjustment (two-sided). Asterisks denote adjusted p thresholds (*p<0.05, **p<0.01, ***p<0.001)

These favorable shifts in the molecular microenvironment directly influenced cellular homeostasis. Total apoptosis increased versus control across all arms (peak 1.29× with BA 2.5 mM). Early apoptosis rose in every group (highest with BA 2.5 mM, 34.17% vs. 27.13%), while late apoptosis was unchanged except for a modest increase with BA 2.5 mM (14.51% vs. 10.66%, also > TA 10 µM). Necrosis remained negligible across conditions (≈ 0.04–0.21%). (Fig. 4A-B).

### Cell Cycle (Flow Cytometry)

All treatments shifted cells toward G0/G1 with reciprocal reductions in S and G2/M versus control (all *p* < 0.01). The strongest effect was with BA 2.5 mM-G0/G1 77.7% vs. 38.6% and G2/M 8.1% vs. 17.1% (also >/< TA 10 µM in pairwise tests), with similar but smaller shifts for TA 10 µM, BA + TA 10 µM, and BA + TA 5 µM. S phase declined across all active arms (~ 20–35% vs. 56.5% control). (Fig. 4C-D).

### Histology (H&E)

**Fig. 5:**
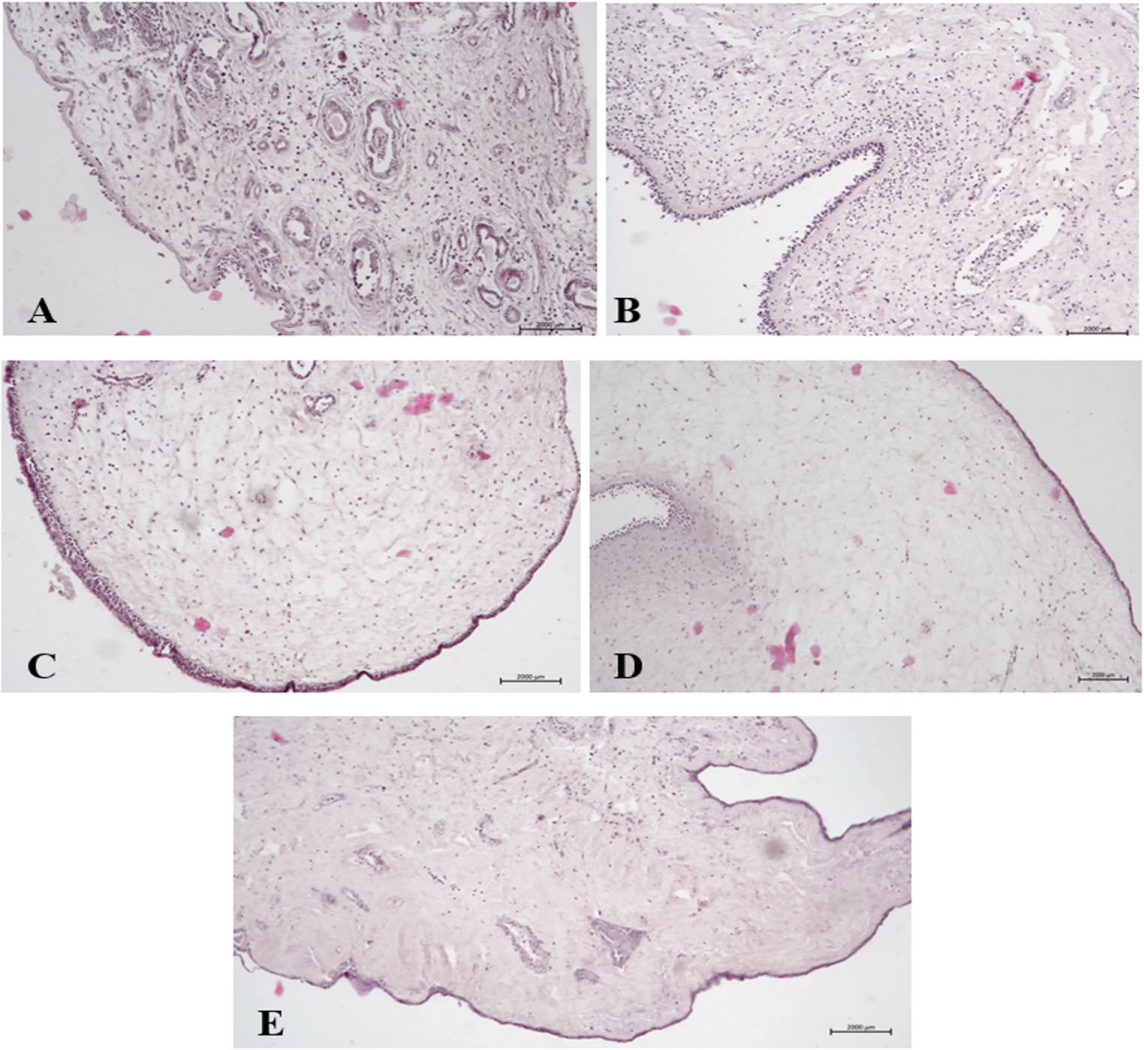
Qualitative H&E (10×) assessment of inflammatory response in nasal polyp tissue. (**A**) Control: diffuse lamina propria infiltration, oedema, and congestion. (**B**) TA 10 µM: decreased cellularity vs. control. (**C**) TA 10 µM + BA 2.5 mM and (**E**) BA 2.5 mM: marked reduction in infiltrates and more uniform stromal matrix. (**D**) TA 5 µM + BA 2.5 mM: lowest cellularity. TA, triamcinolone acetonide; BA, boric acid

Ultimately, the cumulative molecular and cellular effects of reduced oxidative stress, suppressed cytokine signaling, and halted ECM degradation translated into marked improvements at the tissue architecture level. The histological analysis, supported by semi-quantitative data, demonstrated a clear ranking of groups (Table 4). While the control group (A) and the 10 µM T group (B) showed severe inflammatory cell infiltration, a graded reduction was observed in Boron-treated groups. The 5 µM T + 2.5 mM B (D) and 2.5 mM B (E) groups exhibited the most significant decrease in both cellular infiltration and stromal edema (Fig. 5).

**Table 4:** Semi-quantitative histopathological scoring of inflammatory cell infiltration, stromal edema, and vascular congestion in nasal polyp tissues across experimental groups

Groups	Inflammatory Cell Infiltration	Stromal Edema	Vascular Congestion	Total Score
Control	3.0	2.8	2.2	8.0
10 µM TA	3.0	2.2	1.8	7.0
10 µM TA + 2.5 mM BA	1.8	1.2	1.0	4.0
5 µM TA + 2.5 mM BA	1.2	0.8	0.6	2.6
2.5 mM BA	0.8	0.4	0.4	1.6

Scoring was performed on a scale of 0–3: 0 (none), 1 (mild), 2 (moderate), and 3 (severe)

## Discussion

The concurrent improvements observed across oxidative, inflammatory, and ECM markers suggest that BA modulates an integrated pathophysiological network in CRSwNP, rather than acting on isolated pathways. Our findings support the concept of a ‘redox-protease axis,’ wherein the attenuation of the upstream oxidative load by BA is mechanistically linked to the suppression of downstream cytokine signaling. This sequential down-modulation subsequently recalibrates the ECM balance by inhibiting MMP-9 activity, thereby fostering a microenvironment conducive to tissue homeostasis and structural preservation.

### Modulation of the Redox-Protease Axis

The concurrent improvements observed across oxidative, inflammatory, and ECM markers suggest that BA modulates an integrated pathophysiological network in CRSwNP. To the best of our knowledge, this study is the first to delineate the multiplane effects of BA in human CRSwNP explants. Our findings support the concept of a ‘redox-protease axis,’ wherein the attenuation of the upstream oxidative load by BA is mechanistically linked to the suppression of downstream cytokine signaling. This sequential down-modulation recalibrates the ECM balance by inhibiting MMP-9 activity and promoting TIMP-1. By halting pathological ECM degradation, this mechanism represents a crucial step toward restoring epithelial barrier integrity and fostering mucosal repair.

### Steroid-Sparing Strategies

The most clinically important output of this study is the combined effect that boric acid shows with corticosteroids. The observation that BA combined with low-dose TA (5 µM) achieves comparable anti-inflammatory and tissue-protective effects to standard-dose TA (10 µM) strongly demonstrates that BA can be used as a potential “steroid-sparing” agent. Mechanistically, this combined stems from BA’s ability to concurrently target two fundamental pathological axes of CRSwNP: neutralizing reactive oxygen species and inhibiting NF-κB-driven inflammation, which is biologically consistent with its known capability to activate NRF2 [15]. Especially considering that NOX2-derived ROS accumulation and NLRP3 inflammasome activation play a key role in corticosteroid-resistant phenotypes, the targeting of this axis by BA further increases its therapeutic potential [16]. Therefore, this bidirectional mechanism of BA provides a strong rationale for a novel adjuvant therapeutic approach. It is particularly relevant for patient subgroups that respond inadequately to standard steroid therapy due to neutrophilic inflammation and high oxidative load. By targeting these pathways, BA may increase treatment efficacy while reducing the side effects associated with long-term corticosteroid use.

### Cellular Homeostasis and Tissue Architecture

Beyond immunomodulation, our findings demonstrate that BA directly regulates cellular homeostasis in CRSwNP explants. The observed induction of early apoptosis and G0/G1 cell-cycle arrest serves as a targeted counter-intervention to the well-defined hyperproliferative state and accelerated G1/S transition pathology inherent to nasal polyps [17]. This aligns with established literature indicating that BA triggers apoptosis via the BAX/Casp3 pathway in other tissues [18]. Tissue-level effects mirrored these cellular changes; while immune infiltration was markedly reduced in the BA and BA + TA groups, the persistence of infiltrates in the TA-only group underscores the refractory role of IL-5, a cytokine heavily associated with relapse in steroid-resistant subtypes [16]. Consequently, BA emerges as a promising complementary candidate, particularly for the recalcitrant Type-2-high/eosinophilic phenotype. Crucially, this cell-cycle arrest and apoptosis induction did not lead to tissue toxicity. Instead, as confirmed by our histological assessments, it facilitated the clearance of the inflammatory infiltrate and resolved stromal edema, thereby validating the therapeutic efficacy of the BA-mediated redox-protease modulation at the macro-tissue level. Notably, we observed a slight increase in IL-4/IL-13 mRNA levels at 72 h in the BA + T 5 µM arm, despite suppressed protein levels. This mRNA-protein discrepancy is frequently encountered in ex vivo systems. It likely reflects complex post-transcriptional regulation, translational efficiency, or secretion kinetics rather than a true biological anomaly.

## Limitations of the Study

Despite its findings, this study has several limitations. Although the ex vivo explant model preserves native mucosal architecture, it cannot fully recapitulate key in vivo determinants, including systemic immune cell trafficking, mucociliary clearance, bioavailability, and critically nasal-mucosal pharmacokinetics, for which validated human exposure data for boric acid remain unavailable. Accordingly, the selected boric acid concentration should be interpreted as a conservative, tissue-compatible ex vivo exposure rather than an in vivo-equivalent mucosal level. In addition, the 72-hour observation window may not capture delayed post-transcriptional effects or longer-term remodeling. Finally, the present design does not constitute a formal drug drug interaction experiment (i.e., a factorial dose–response matrix). Therefore, the combined BA + TA findings should be interpreted as an enhanced combined effect and a potential steroid-sparing signal, rather than definitive pharmacological synergy. Future work should integrate dynamic mucosal platforms under physiological flow, nasal mucosa specific dose–response and PK/PD-oriented studies, and early phase dose finding clinical trials to define translational exposure ranges and to formally evaluate drug–drug interactions using quantitative interaction modeling (e.g., isobologram or combination index).

## Conclusion

In conclusion, our ex vivo investigation demonstrates that BA effectively suppresses type 2 inflammation, reduces oxidative stress, and attenuates pathological tissue remodeling in human nasal polyp explants. Mechanistically, BA orchestrates this multidimensional therapeutic response by modulating the “redox-protease axis” concurrently inhibiting upstream oxidative load and downstream inflammatory signaling to recalibrate the ECM balance, halt tissue degradation, and promote cellular homeostasis. Clinically, the observed combined efficacy between BA and low-dose TA positions BA as a highly promising “steroid-sparing” agent. While these findings are encouraging, future studies utilizing dynamic mucosal platforms and early-phase clinical trials are warranted to validate BA’s in vivo pharmacokinetics and its ultimate utility as an innovative adjuvant in the management of recalcitrant CRSwNP.

## Data Availability

No datasets were generated or analysed during the current study.
