# Correction: Comparative Evaluation of Therapeutic Effects of Boric Acid and Triamcinolone in Nasal Polyp Culture

**DOI:** 10.1007/s12011-026-05279-z

**Published:** 2026-08-05

**Authors:** Nurullah Türe, Meliha Koldemir Gündüz, Güllü Kaymak, Ayşe Koçak Sezgin, Mehmet Varol

**Affiliations:** 1 Department of Otorhinolaryngology, Kütahya Health Sciences University, Bahçelievler Mahallesi Başyiğit Sokak Semakent Sitesi A Blok no:29/8, Kütahya, Türkiye; 2 Department of Basic Engineering Sciences, Kütahya Health Sciences University, Kütahya, Türkiye; 3 Department of Medical Services and Techniques, Kütahya Health Sciences University, Kütahya, Türkiye; 4 Department of Medical Biochemistry, Kütahya Health Sciences University, Kütahya, Türkiye; 5 Department of Otorhinolaryngology, Kütahya City Hospital, Kütahya, Türkiye

**Correction to: Biological Trace Element Research (2026) 204:5404–5418**

**https://doi.org/10.1007/s12011-026-05040-6**

The original version of this article contained a mistake. 

In "Materials and Methods – Study Design and Ethical Approval", the sentence: 

"This single-centre prospective cohort, incorporating an ex vivo, patient-matched randomized block experiment, was conducted in the XXX Health Sciences University (XHSU)." should read: "This single-centre prospective cohort, incorporating an ex vivo, patient-matched randomized block experiment, was conducted at Kütahya Health Sciences University (KHSU)."

The following sentence should be added to the Funding section, alongside the existing statement on open access funding provided by TÜBİTAK: "This research was supported by the Scientific Research Committee of Kütahya Health Sciences University (Project No. TSA-2024-190)."

The original article has been updated.

